# The Bidirectional Link Between RNA Cleavage and Polyadenylation and Genome Stability: Recent Insights From a Systematic Screen

**DOI:** 10.3389/fgene.2022.854907

**Published:** 2022-04-28

**Authors:** Stefano Spada, Brian Luke, Sven Danckwardt

**Affiliations:** ^1^ Posttranscriptional Gene Regulation, University Medical Centre Mainz, Mainz, Germany; ^2^ Institute for Clinical Chemistry and Laboratory Medicine, University Medical Centre Mainz, Mainz, Germany; ^3^ Centre for Thrombosis and Hemostasis (CTH), University Medical Centre Mainz, Mainz, Germany; ^4^ Institute of Molecular Biology (IMB), Mainz, Germany; ^5^ Institute of Developmental Biology and Neurobiology (IDN), Johannes Gutenberg University, Mainz, Germany; ^6^ German Centre for Cardiovascular Research (DZHK), Berlin, Germany; ^7^ Centre for Healthy Aging (CHA) Mainz, Mainz, Germany

**Keywords:** cleavage and polyadenylation, DNA damage response, genome integrity, cancer, alternative polyadenylation, systematic screening, resillience, aging

## Abstract

The integrity of the genome is governed by multiple processes to ensure optimal survival and to prevent the inheritance of deleterious traits. While significant progress has been made to characterize components involved in the DNA Damage Response (DDR), little is known about the interplay between RNA processing and the maintenance of genome stability. Here, we describe the emerging picture of an intricate bidirectional coupling between RNA processing and genome integrity in an integrative manner. By employing insights from a recent large-scale RNAi screening involving the depletion of more than 170 components that direct (alternative) polyadenylation, we provide evidence of bidirectional crosstalk between co-transcriptional RNA 3′end processing and the DDR in a manner that optimizes genomic integrity. We provide instructive examples illustrating the wiring between the two processes and show how perturbations at one end are either compensated by buffering mechanisms at the other end, or even propel the initial insult and thereby become disease-eliciting as evidenced by various disorders.

## DNA Damage Regulation

The exposure of cells to exogenous or endogenous stresses (such as UV-light, radiation, certain pharmaceuticals, oxidative agents as well as replication or transcription errors) can result in structural alterations of the DNA. To maintain the integrity of the genome, cells are equipped with intricate (and sometimes redundant) molecular networks that are ready to both detect and correct DNA damage. These networks take part in a protective cellular program known as DNA damage response (DDR) that, beyond merely detecting the damage, is also responsible for activating cellular checkpoints, regulating gene expression, repairing the lesions, and inducing apoptosis, in case the damage exceeds the cell-intrinsic repair capacity. The coordinated sequence of these events involves dedicated components, including DNA damage sensors (such as the apical kinases ATM or ATR and the DNA-coating counterparts MRE11-RAD50-NBS1 complex and RPA) as well as effector proteins (including the checkpoint kinases Chk1 and Chk2), which in turn arrest the cell cycle until the damage is repaired. Although there is still much to be learned, the main processes underlying the DDR are understood in great detail (Zhou and Elledge, 2000; Ciccia and Elledge, 2010; Pilié et al., 2019). It is becoming increasingly apparent however, that RNA regulation is adding a layer of complexity to the DDR in terms of RNA transcription, RNA turnover, and post-transcriptional modifications. Indeed, RNA metabolism is emerging as a critical contributor to genome integrity, starting from early observations that steady-state transcripts levels are decreased upon DNA damage (Lenzken et al., 2013). Several layers of transcriptome regulation are involved in this effect. For example, initiation of transcription has been reported to be repressed in response to UV light-induced damage due to sequestration of the TATA-binding protein from the preinitiation complex (Vichi et al., 1997) and depletion of the initiating hypophosphorylated form of RNA polymerase II (RNAPII) (Rockx et al., 2000). UV light has also been shown to increase phosphorylation of the C-terminal domain (CTD) of RNAPII, leading to a decrease in elongation rates along with changes in alternative splicing (Muñoz et al., 2010). But also pre-mRNA 3′end processing has been linked to mRNA stability during DDR (Kleiman and Manley, 2001). Processing of the RNA 3′end is a crucial feature of most genes and impinges on translocation of the mRNA from the nucleus to the cytoplasm, transcript stability, and ultimately protein output (Danckwardt et al., 2008). It is therefore not surprising that communication between the 3′end processing machinery and the DDR exists to ensure optimal RNA processing and hence genome stability.

The present work focuses on the bidirectional connection between DDR and 3′end processing of genes, i.e. how the DDR affects 3′ end processing and how this, in turn, impinges on the DDR. Apart from illustrating a few well-known examples that connect DDR with 3′end processing, we are also showcasing insights from a recently performed large-scale screening (Ogorodnikov et al., 2018) suggesting that alterations of 3′end processing that normally play a role in damage repair may, under certain circumstances, also propel the DNA damage.

## DDR Alterations Affect 3′End Processing Events

**FIGURE 1:**
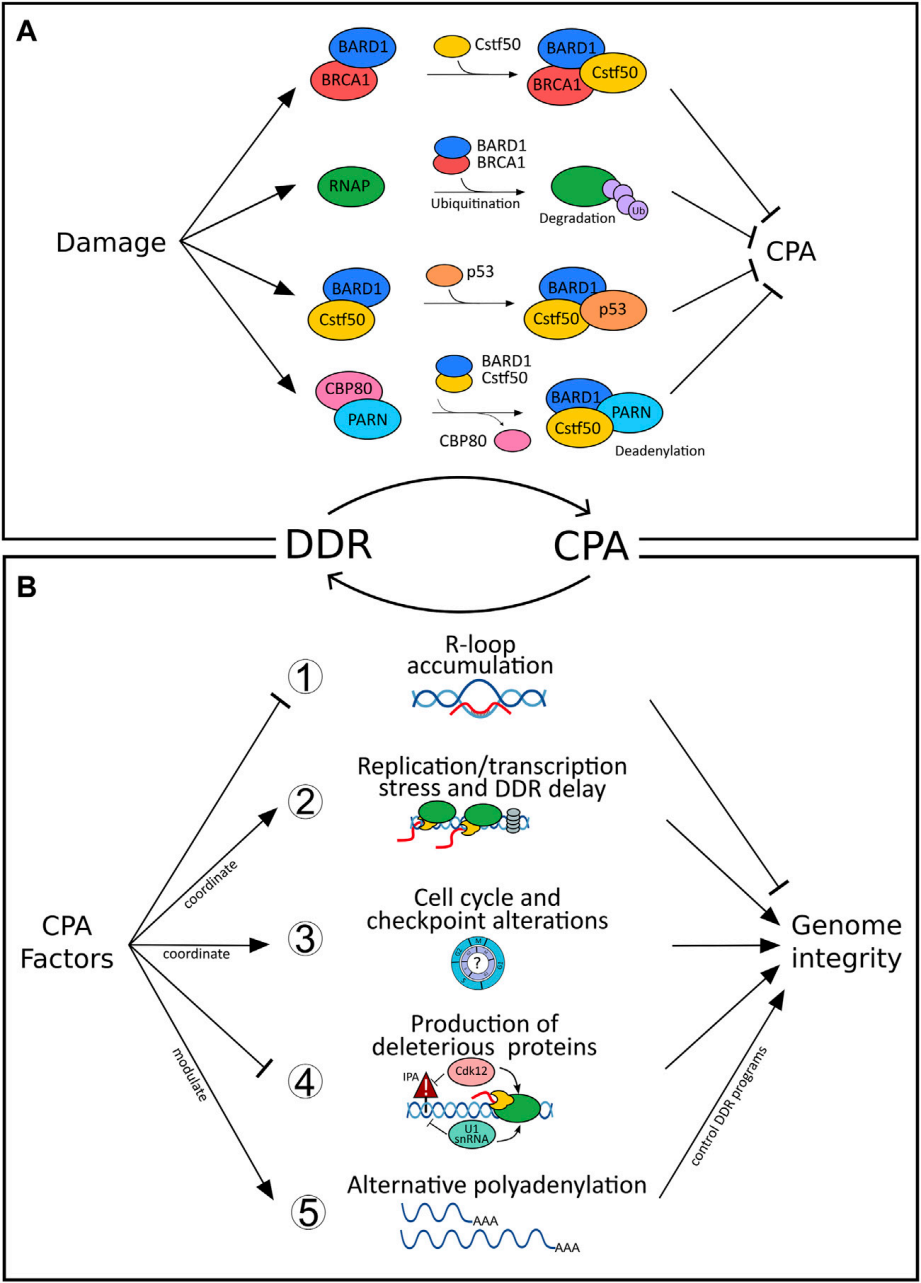
Bidirectional connections between DNA damage response (DDR) and cleavage and polyadenylation (CPA). **(A)** Inhibition of cleavage and polyadenylation (CPA) as a response to DNA damage fosters repair of the genomic lesions and prevents the release of defective transcripts. For example, CstF-50/BARD1/BRCA1-complex formation after UV irradiation blocks RNA 3′ endonucleolytic cleavage (Kleiman and Manley, 2001) and results in RNAP II ubiquitination and proteasomal degradation (Kleiman et al., 2005). Similarly, binding of the p53-BARD1 complex inhibits the 3′end cleavage activity of CstF-50 (Nazeer et al., 2011). The interaction between CstF-50 and PARN shifts the balance between polyadenylation and deadenylation, ultimately controlling the level of mature RNAs in response to DNA damage (Cevher et al., 2010). CBP80, which inhibits deadenylation through PARN binding, dissociates from PARN upon damage and activates cap-dependent deadenylation resulting in reduced levels of total mRNAs (Balatsos et al., 2006). In contrast, **(B)** CPA can impair genome integrity when deregulated (further details see text, Supplementary Table S1 and Figure 2). For example, (1) loss of function of select CPA components (such as PCF11, CLP1, FIP1L1, CFT2, WDR33) impairs genome integrity by resulting in R-loop formation (Stirling et al., 2012; Teloni et al., 2019). (2) Transcription and replication are coordinated to prevent collision between both machineries and prevent genomic instability (Gaillard and Aguilera, 2014). CFIm depletion impairs transcription termination, which interferes with replication and results in the delay of DDR (Gaillard and Aguilera, 2014) as well as increased sensitivity to UV light (Mirkin et al., 2008). (3) Alterations of CPA components result in cell cycle and checkpoint alterations; for example, CstF64 deficiency results in G0/G1 arrest (Takagaki and Manley, 1998) or loss of symplekin in a G2/M arrest (Ruepp et al., 2011). (4) Cdk12 suppresses intronic polyadenylation (IPA) and thereby fosters the production of full-length gene products, which for example affects many homologous recombination genes. Intronic alternative polyadenylation resulting from U1snRNA downregulation after UV (Devany et al., 2016) alters IPA of genes involved in the UV-response (such as POLR2A and CDKN1A). (5) >70% of all genes are affected by alternative polyadenylation (APA) resulting in functional diversity (Nourse et al., 2020). CPA factors pervasively control APA (Marini et al., 2021) and thereby drive programs involved in the DDR (Figure 2; Supplementary Table S1).

In addition to capping and splicing, almost all transcripts in eukaryotes undergo further processing at the RNA 3′end. For most genes, this involves endonucleolytic cleavage and non-templated polyadenylation (CPA) before the mature RNA can be exported into the cytoplasm (Danckwardt et al., 2008) (and refs. therein). As CPA controls almost all genes, regulation of CPA has evolved as an important layer of gene expression. Under damaging conditions, CPA plays a role in RNA surveillance, which prevents the “release” of inappropriate and potentially deleterious transcripts (Nourse et al., 2020). CPA is carried out by a multi-subunit complex involving over 80 trans-acting proteins organized in four core protein subcomplexes, the *cleavage and polyadenylation specificity factor* (CPSF), *cleavage factor I* (CFI), *cleavage factor II* (CFII), and the *cleavage stimulation factor* (CstF) (Proudfoot, 2016). The recruitment of these multimeric complexes to dedicated, but largely poorly conserved, processing sites (Gruber et al., 2016) ensures that 3′end processing of the nascent transcript occurs in a timely manner and at the right position (Danckwardt et al., 2008). 3′end processing involves an intricate interaction between these complexes (and subcomponents) and RNA polymerase II (RNAP II) (Proudfoot, 2016). Surprisingly, the CPA machinery does not exclusively associate with components involved in the processing of the pre-mRNA but also interacts with certain DDR proteins (Figure 1A). For example, CstF-50, a CstF subunit, interacts with BARD1 and BRCA1 (Kleiman and Manley, 2001), both of which have established functions in DNA repair and checkpoint controls. Upon UV irradiation, levels of CstF-50/BARD1/BRCA1 complexes have been shown to increase (Kleiman and Manley, 2001) leading to a reduction of RNA 3′ endonucleolytic cleavage by direct interaction between CstF-50 and the BARD1 subunit of the BRCA1/BARD1 heterodimer (Kleiman and Manley, 1999). Moreover, BRCA/BARD1 targets the hyperphosphorylated RNAP II for ubiquitination and ultimately proteasomal degradation, inhibiting the coupled transcription-RNA processing machinery and facilitating repair (Kleiman et al., 2005). Consistent with this bridging function of CstF between CPA and DDR, depletion of CstF proved to enhance UV sensitivity, prevent DNA repair, and lead to cell-cycle arrest and apoptosis in B Cells and HeLa cells (Takagaki and Manley, 1998; Mirkin et al., 2008). Similarly to the BRCA1-BARD1-CstF-50 complex, CstF-50 was also shown to bind to the p53-BARD1 complex *in-vitro,* which results in an inhibition of the 3′end cleavage (Nazeer et al., 2011). Thus, modulating CPA appears to be an important target of the DDR, which could increase survival in multiple ways: i) in preventing damaged transcripts from producing toxic proteins and ii) to promote efficient repair of the genomic lesion (further detailed below). In addition, shifting the balance between polyadenylation and deadenylation can also control the total level of (preexistent) mature RNAs in response to DNA damage, as illustrated by the interaction between CstF-50 and Poly(A)-Specific Ribonuclease (PARN) (Cevher et al., 2010). Here, the Nuclear cap-binding protein subunit 1 (CBP80), which inhibits deadenylation through PARN binding, dissociates from PARN and thereby activates cap-dependent deadenylation resulting in reduced levels of total mRNAs (Balatsos et al., 2006). Additionally, PARN can directly decrease the stability of the p53 mRNA in non-stress conditions through ARE sequence present in the 3′UTR of p53 mRNA (Devany et al., 2013). Under DNA damage conditions, p53 protein accumulation allows its association to, and activation of, PARN ultimately decreasing the expression of target mRNAs in the p53-dependent DDR pathway.

Taken together, these mechanisms involving core CPA factors during DDR evidence an important role of 3′end processing in minimizing the risk of generating inadequate polyadenylated mRNA isoforms resulting from damaging conditions.

## Additional Factors Modulating CPA During DDR

Although evidence linking 3′end processing and DDR exists, the underlying molecular connections are still poorly defined. Insights may emerge through the realization that several factors engaged in the DDR are also a part of the 3′end processing complex (Shi et al., 2009). This is indeed the case for the serine/threonine-protein kinase complex DNA-PKcs-Ku70-Ku86, although its direct role in the phosphorylation of CPA components has not yet been fully clarified (Ariumi et al., 1999). DNA-PK can phosphorylate the Poly (ADP-ribose) polymerase 1 (PARP1), which is both functionally associated with CPA and involved in the DDR. RBBP6, an E3 ubiquitin ligase initially discovered to interact with p53 (Simons et al., 1997), has also been found as part of the 3′end processing machinery. Mpe1, the yeast homolog of the E3 ubiquitin-protein ligase RBBP6, is able to ubiquitinate the poly(A) polymerase PAP1 (Lee and Moore, 2014), suggesting that a possible connection between ubiquitination and CPA for mammal cells may also exist. Corroborating this idea, some RBBP6 protein isoforms alter polyadenylation signal selection and promote alternative polyadenylation at distal signals throughout the genome (Di Giammartino et al., 2014). Furthermore, the tumor suppressors CSR1 (cellular stress response 1) and CDC73 have been found to modulate 3′end processing via inducing CPSF3 translocation from the nucleus to the cytoplasm and consequently inhibiting CPA or facilitating association of 3′end processing factors with actively-transcribed chromatin, respectively (Rozenblatt-Rosen et al., 2009; Zhu et al., 2009).

## Selective 3′End Processing of DDR Genes

The efficient response to DNA damage requires the expression of multiple DDR genes for the repair process to begin. DDR expression must occur, despite the above-described global inhibition of transcription and 3′UTR processing (Figure 1A). Due to this paradoxical scenario, compensatory mechanisms must be in place to allow selective transcription and escape from the global 3′UTR processing inhibition following DNA damage. In fact, increased transcription for some DDR-responsive genes has been observed (Christmann and Kaina, 2013) and it is conceivable that their upregulation compensates for the inhibition of processing. However, specific regulatory features have also been found to facilitate the 3′end processing of DDR genes. The intrinsic strength of polyA signals (PAS), downstream sequence elements (DSE), and upstream sequence elements (USE) appear to be enhanced in DDR genes fostering effective 3′end processing even in conditions favoring low-levels of CPA complex formation. RNA secondary structures are involved, as exemplified by the tumor suppressor p53 (Decorsière et al., 2011). The p53 pre-mRNA is capable of bypassing the general 3′processing inhibition upon UV-C stimulation via interaction with the ribonucleoproteins hnRNP H/F after an RNA helicase, DHX36, unwinds a G-quadruplex structure downstream of a p53 polyadenylation site to allow access of hnRNP H/F promoting effective 3′end processing (Newman et al., 2017).

Apart from processes acting in cis, 3′end processing of DDR genes can also be facilitated through determinants in trans. For instance, select genes that belong to the p53 pathway bypass the requirement for RNAP II phosphorylation on Ser2, which is usually required for proper 3′end processing (Gomes et al., 2006). However, other early response genes, such as c-fos and junB, are dependent on Ser2 phosphorylation, suggesting that different compensatory mechanisms might exist for different stress stimuli (Fujita et al., 2008). Other types of RNA polymerases can also participate in DDR regulation. This is the case of the non-canonical Star-PAP and its ability to bind directly the pre mRNA (Laishram and Anderson, 2010). Upon stress induction, Star-PAP can regulate 3′end processing in a gene- and condition-specific manner. As an example, etoposide treatment induces Star-PAP binding to the proapoptotic gene Bcl-2 interacting killer (BIK) mRNA (Li et al., 2012) while other stressors (oxidative stress) induce Star-PAP regulation of the cytoprotective heme oxygenase HO-1 (Mellman et al., 2008) and quinone oxidoreductase NQO1 (Gonzales et al., 2008). In addition, Star-PAP can also promote alternative polyadenylation (APA), a pervasive mechanism of transcriptome diversification (Ogorodnikov et al., 2016) (further detailed below), of the tumor suppressor PTEN (Li et al., 2017) mediating increase of PTEN protein upon DNA damage. This further supports the involvement of CPA in the regulation of damage-responsive mRNAs. But also factors not belonging to the core CPA machinery are known to modulate APA after DNA damage. The cyclin-dependent kinase CDK12, beyond maintaining RNAP II processivity through its phosphorylation, is also implicated in various pre-mRNA processing mechanisms. Transcript isoforms of the DNA damage response activator ATM are regulated via modulation of alternative last exon (ALE) inclusion by CDK12 (Tien et al., 2017). Further, CDK12 is known to protect from premature CPA and loss of expression of long genes, including those participating in the DDR (Krajewska et al., 2019). p38 MAPK, another kinase known to promote cell survival under damaging conditions (Phong et al., 2010), modulates polyadenylation after (genotoxic) stress in prothrombin and several other cancer-associated genes including BCL2L2, OTUD7B, PDCD10, PDGFRA as well as KIN with a role in DNA replication and the cellular response to DNA damage (Danckwardt et al., 2007; Danckwardt et al., 2011). Here, select trans-acting RNA-binding proteins (U2AF35, U2AF65, FBP2, FBP3) bind to defined RNA elements (USEs) in a phosphorylation-dependent, antagonistic manner to confer specificity of stress-dependent CPA modulation (Danckwardt et al., 2011). This example illustrates how determinants in cis and trans can co-cooperate resulting in “dual authentication” of condition-dependent selective 3′end RNA processing.

Ultimately, regulation of DDR genes can also occur through mRNA decay mechanisms, which are usually strongly determined by the 3′UTR architecture. One such example is provided by the recruitment of RNA-binding proteins (RBPs) to AU-rich elements (ARE) typically residing in this region. These interactions can either stabilize or destabilize transcripts. For instance, the ARE-binding protein AUF1 can destabilize the growth arrest and DNA damage-inducible gene GADD45a under healthy conditions (Lal et al., 2006). Here, AUF1 competes with the Poly(A) binding protein PABP (Sagliocco et al., 2006) for binding to the polyA tail and promotes recruitment of exosome and proteasomal degradation. Upon genotoxic stress, however, AUF1 dissociates resulting in prolonged GADD45α mRNA half-life. Similarly, interaction with the RBPs TTP and KSRP, known to relay signals of genotoxic stress (Briata et al., 2016; Lee et al., 2020)**,** leads to rapid degradation of ARE-containing target mRNAs through recruitment of deadenylases (Winzen et al., 2007; Clement et al., 2011). Conversely, binding of HuR fosters stabilization of transcripts involved in carcinogenesis, cell proliferation and survival, and oxidative and genotoxic cellular response (Westmark et al., 2005; Li et al., 2018). Collectively, these findings document that the processing of DDR genes can be regulated on various layers to likely accommodate the specific needs under conditions of genotoxic stress.

## CPA Factors can Modulate the DDR

Although mounting evidence suggests that 3′end processing may be a key regulator taking part in genome stability and the damage response, the reciprocal role of CPA components in modulating the DDR remains elusive (see above). Evidence for a critical function of CPA for the maintenance of genome integrity, independent from a primary genotoxic insult, is provided by R-loops (Figure 1B). R-loops are three-stranded structures composed of an RNA-DNA hybrid and a displaced single-stranded DNA (ssDNA) (Crossley et al., 2019; García-Muse and Aguilera, 2019). When R-loops are not removed in a timely manner, they can lead to compromised genome integrity. R-loops are abundant in the proximity of polyadenylation sites and correlate with efficient termination of transcription (Sanz et al., 2016). The coordinated co-transcriptional processing and packaging of the nascent transcript into “inert” ribonucleoprotein particles (RNPs) ensures that R-loops occur in a scheduled manner preventing the formation of otherwise deleterious RNA-DNA hybrid structures (Danckwardt et al., 2008). Events that perturb the coordinated co-transcriptional processing result in unscheduled R-loop accumulation, which can lead to replication-associated DNA damage. A loss-of-function screening study performed in budding yeast highlighted seven essential protein components of 3′end processing machinery (including PCF11, CLP1, FIP1L1, and CFT2) to maintain genome integrity by suppressing R-loop formation (Stirling et al., 2012). In another study, deregulation of WDR33, a component of the core CPA machinery, impaired cleavage of nascent pre-mRNA leading to R-loop accumulation and slowing of the replication fork during the S phase (Teloni et al., 2019). Additional factors, localized at the RNA 3′end and cooperating in transcription termination demonstrate the same functional outcome, as in the case of loss of function of Rtt103, yeast homolog of RPRD1B, (Stirling et al., 2012), Sen1 (senataxin) (Mischo et al., 2011) and XRN2 (Morales et al., 2016). Taken together, the evidence is consistent with findings regarding the harmful effects of (unscheduled) R-loops (beyond their physiological role in transcription termination) and supports a novel contribution of CPA in promoting genome integrity (Figure 1B).

CPA regulated replication stress can however also take other forms (beyond R-loops defects) and different players come into action. For instance, CFIm depletion mediates a delayed activation of DNA damage checkpoint signaling and RNA polymerase II degradation following DNA damage (Gaillard and Aguilera, 2014) as well as increased sensitivity to UV light (Mirkin et al., 2008). Similarly, depletion of CstF64, leads to destabilization of the entire Cstf complex, rendering cells deficient in recovery from UV treatment and in the repair of the UV-induced DNA lesions (Mirkin et al., 2008). CstF64 deficient cells accumulate in the G0/G1 stage of cell cycle (Takagaki and Manley, 1998). Alterations in cell cycle progression are also seen upon depletion of symplekin (Ruepp et al., 2011), which may affect repair pathway decisions. Similarly, depletion of CPSF73 and CPSF100, with resulting effects on symplekin expression, affected histone pre-mRNA processing (Sullivan et al., 2009). Finally, CstF2tau has recently been shown to control the abundance of snRNAs (through alternative oligoadenylation) resulting in alternative splicing of several RNAs including the histone deacetylase HDAC2 (Kargapolova et al., 2017), a critical component in the maintenance of genome stability (Miller et al., 2010; Dovey et al., 2013).

The role of CPA factors in safeguarding genome integrity has also been documented beyond their function in RNA processing. After ubiquitin-mediated degradation of RNAP II, chromatin must be made accessible to allow the repair machinery to access the lesion. Here, co-recruitment of CstF-50 with the ubiquitin escort factor p97 contributes to the displacement of ubiquitinated histones H2A, H2B, and nucleosome remodeling (Fonseca et al., 2018). Moreover, Cdk12 has recently been shown to globally suppress intronic polyadenylation thereby fostering the production of full-length gene products (Figure 1B). This also affects many homologous recombination genes, and accordingly Cdk12 loss of function mutations frequently found in tumors globally impair genome integrity (Dubbury et al., 2018).

These findings indicate that individual CPA components (and associated factors) can likely impair central processes that are directly involved in the suppression or the propagation of genotoxic stress. However, what is the evidence that CPA components can be more globally linked to the surveillance of genome integrity?

## Global Insights Linking DDR With Alternative Cleavage and Polyadenylation

Early reports on UV-irradiated sun-damaged fibroblasts suggested a link between the usage of different polyA signals (PAS) and genotoxic stress (Schwartz et al., 1998). The regulation of PAS choice in response to damaging agents seems to depend on the trigger as well as on the cell type. Intronic alternative polyadenylation, mediated by U1snRNA downregulation was observed upon UV treatment of mammalian cells (Devany et al., 2016). Topoisomerase inhibitors, on the other hand, promoted alternative last exons (ALE) through APA (alternative polyadenylation) (Dutertre et al., 2014). In yeast, the same UV treatment led to a global lengthening of transcripts (Graber et al., 2013), also observed on cells after treatment with anisomycin (Hollerer et al., 2016). In contrast, the damaging agent arsenite led to transcript shortening and preferential degradation of species with long 3′ends (Zheng et al., 2018).

**FIGURE 2:**
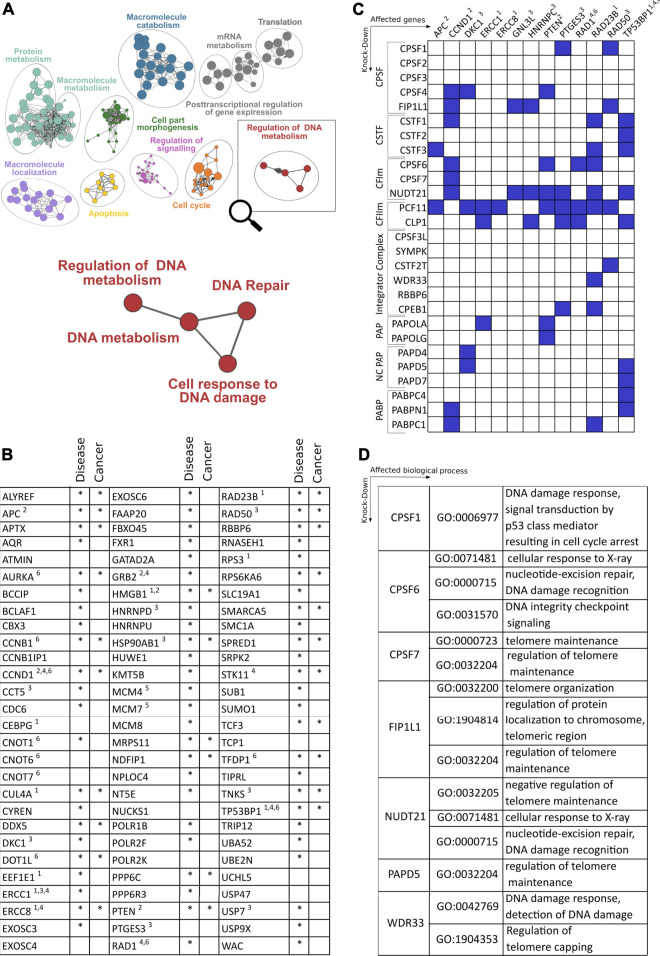
Role of cleavage and polyadenylation factors (CPA) on genes and pathways involved in DNA damage response. **(A)** Enriched GO terms based on genes that show alterations of polyadenylation after depletion of the CFIm component PCF11 (Ogorodnikov et al., 2018; Marini et al., 2021). **(B)** Close-up of nodes with functional enrichment centering around regulation of DNA metabolism based on 83 genes regulated upon depletion of PCF11. The table depicts the identity of altered genes and their involvement in disease including cancer (selected entities of DDR response mechanisms highlighted are: 1. DNA repair/nucleotide excision, 2. ATM-dependent DNA response, 3. Telomer regulation, 4. Response to X-ray, 5. Double strand breaks, 6. DNA damage checkpoint response). **(C)** Matrix of selected genes (*x*-axis; functional assignment see legend in **(B)**) involved in DDR with significant alterations of polyadenylation after depletion of canonical and non-canonical 3′end processing factors (*y*-axis; blue boxes indicate significant changes; data obtained from TREND-DB (Marini et al., 2021) covering a large scale RNAi screening (Ogorodnikov et al., 2018) coupled to transcriptome-wide interrogation of alterations in polyadenylation by TRENDseq (Ogorodnikov and Danckwardt, 2021); further details see text). **(D)** Gene Ontology (GO) enrichment of processes centering around DDR upon depletion of CPA factors indicated (Supplementary Table S1 provides further details on DDR genes affected by alternative polyadenylation).

Recently, diversification of the transcriptome at the 3′end by APA emerged as a pervasive and evolutionarily conserved layer of gene regulation (Derti et al., 2012). It affects more than 70% of all genes, resulting in transcript isoforms with distinct 3′end termini. APA thereby considerably expands the diversity of the transcriptome 3′end (TREND). This leads to mRNA isoforms with profoundly different physiological effects, by affecting protein output, production of distinct protein isoforms, or modulating protein localization (Mayr, 2017). As APA is globally regulated in various conditions, including developmental and adaptive programs [with perturbations resulting in numerous disorders (Nourse et al., 2020)], it is tempting to speculate that APA may also modulate, respond and contribute to perturbations of DNA damage and its resolution. Interrogating the dynamic APA landscape may thus provide further insights into the functional connection between the CPA machinery and DNA damage. A recent large scale RNAi screening based on the depletion of >170 CPA components and associated factors involved in numerous facets of RNA metabolism showed, on a genome-wide level, how individual (CPA) factors affect the APA landscape (Ogorodnikov et al., 2018), including the resulting effects on gene ontologies (Marini et al., 2021). While CPA components pervasively regulate APA [with key components, namely NUDT21, CPSF6 and PCF11, affecting the largest proportion of genes (Marini et al., 2021)], a significant proportion of APA is controlled by components involved in transcription and other co- and post-transcriptional events (e.g., splicing and RNA turnover) or epigenetic modification (Ogorodnikov et al., 2018). Interestingly, this screening also identified APA regulation to be caused by factors involved in genome surveillance or known to drive tumor-suppressive programs (e.g., TP53), as well as other processes involved in the coupling between oncogenic signals and 3′end processing (such as BARD1, see above) (Marini et al., 2021). Corroborating the role of CPA factors in DDR, alterations of expression levels of CPA components have already been evidenced in cell cycle processes and cancer biology. For instance, NUDT21 levels were found to be downregulated in glioblastoma tumors, where the resulting 3′UTR shortening causally led to enhanced cellular proliferation and tumorigenicity, probably through the upregulation of growth-promoting factors, such as cyclin D1 (Masamha et al., 2014). In agreement, PAK1, which recently emerged as a component of the DDR (Pérez-Yépez et al., 2018), was found to be a downstream target of NUDT21, serving as a predictive prognostic marker for glioblastoma patients (Chu et al., 2019). Contrary to NUDT21 downregulation, CPSF6 or CSTF2 were shown to be upregulated in hepatocellular carcinoma and urothelial carcinoma respectively (Chen et al., 2018; Tan et al., 2021). This in turn correlated with the upregulation of NQO1 and RAC1 through the favorable usage of its proximal 3′UTR poly(A) promoting tumor formation and progression. Further, PCF11 has previously been shown to control pathways (such as EIF2 and IGF1) converging on WNT-signaling (Ogorodnikov et al., 2018), functionally associated with DNA damage (Karimaian et al., 2017; Zhao et al., 2018; Pasadi and Muniyappa, 2020). Interestingly PCF11 constitutes a central component shaping transcriptome 3′end diversity. At the same time, it also represents a developmental switch that directly links RNA 3′end processing with aberrant development and tumor formation (Ogorodnikov et al., 2018). Persistently high levels of PCF11 expression in the postnatal period arrest neuronal precursors in an immature state and ultimately give rise to neuroblastomas, the most common solid tumor in children (Maris, 2010). While high-level PCF11 determines a fatal disease progression, low levels of PCF11 instead associate with favorable outcome and spontaneous tumor regression (Ogorodnikov et al., 2018). The contribution of PCF11 to APA-mediated DDR is seemingly noticeable when taking a closer look into which biological processes are modulated in a PCF11-dependent manner (http://shiny.imbei.uni-mainz.de:3838/trend-db/) (Marini et al., 2021) (Figure 2A). The DNA metabolism cluster, defined by 83 significantly APA-affected genes, falls into various enriched subsets of DNA metabolism including *DNA repair* and *cell responses to DNA damage*. Accordingly, the vast majority of all those genes are associated with various disorders (mainly developmental) but also with other entities including cancer. This corresponds to the previously made observations indicating a central role of PCF11 for differentiation/dedifferentiation and developmental programs (Ogorodnikov et al., 2018; Kamieniarz-Gdula et al., 2019).

As one would expect based on the pervasive function of several CPA components for APA modulation (Marini et al., 2021) and the resulting functional impact on protein output (Ogorodnikov et al., 2018), PCF11 does not only control APA of critical genes involved in the DDR (encompassing a wide variety of functional categories, including DNA damage checkpoint responses, DNA repair, nucleotide excision, ATM-dependent DNA response, telomere function, response to X-ray and double-strand breaks). In fact, albeit to variable extent, depletion of almost all CPA components results in the regulation 3′end processing of critical regulators involved in several aspects of DDR (Figure 2C). While 3′end processing of some of these DDR components appears to be selectively regulated (e.g., the CSA Ubiquitin Ligase Complex Subunit ERCC Excision Repair 8 (ERCC8), ERCC1, or the tumor suppressor adenomatosis polyposis coli (APC)), others tend to be more broadly controlled by several factors of the CPA machinery. Interestingly this includes central genes with known functions in nucleotide excision, DNA damage checkpoint- and ATM-dependent DNA damage response (CCND1, PTEN, RAD23B, TP53BP1). This suggests that modulation of RNA 3′processing can directly influence several components involved in the maintenance of genome integrity. Accordingly, beyond PCF11 (Figures 2A,B), the functional enrichment upon depletion of further CPA components (CPSF1, CPSF6, CPSF7, FIP1L1, NUDT21, PAPD5 and WDR33) also reveals significantly enriched GO terms that are associated with various aspects of DNA damage control and repair (Figure 2D). This suggests that mechanisms have evolved that directly link co-transcriptional processing of pre-mRNAs to genome integrity and support a critical function of APA as a pervasive and evolutionarily conserved layer of gene regulation in this context (Figure 2; Supplementary Table S1).

An interesting, yet to be explored, hypothesis arising from these findings is that such a coupling could serve as a buffering system conferring robustness to biological systems. This would ensure that the otherwise detrimental consequences arising from aberrant RNA processing (such as R-loop formation) result in the induction of compensatory mechanisms governing genome integrity (Ngo et al., 2021); Figure 1B), and vice versa, or result in apoptosis of affected cells when a certain threshold is exceeded. Alternatively, aberrant processing could also initiate (and propagate) a failure of genome surveillance, and thereby aggravate a phenotype resulting from initially (minor) perturbations of 3′end processing. As such, the frequently observed perturbations of APA in cancer deserve attention (Nourse et al., 2020). They can act as non-genomic drivers of cancer (Ogorodnikov et al., 2018) and may also result in consecutive downstream perturbations of mechanisms involved in the surveillance of genome integrity (such as WNT-, EIF2- or IGF1-signaling (Ogorodnikov et al., 2018)) with established roles in genome surveillance (Wek et al., 2006; Vermeij et al., 2016; Pasadi and Muniyappa, 2020), eventually perpetuating and even aggravating the initial disease phenotype. Aberrant 3′end processing could thereby contribute to the clonal evolution of tumor lesions (Hugo et al., 2015). Vice versa, APA alterations could also reflect the consequences of impaired DDR with potentially both ends, either aggravating the resulting effects further or resulting in responses that ultimately help the cell to (sense and) repair the mutation and thereby resolve the genomic instability (Figures 1A,B).

Collectively, CPA components that mediate RNA 3′processing and shape the transcriptome diversity are thus emerging as important pillars in the maintenance of genome integrity and vice versa. In view of the emerging opportunities to interfere with CPA (and APA) in a targeted manner (Nourse et al., 2020) further functional characterization of the bidirectional link between both processes may also open hitherto novel untapped therapeutic avenues.

## Outlook

Tools capable of elucidating polyadenylation and involved components in a transcriptome-wide manner (Kargapolova et al., 2017; Ogorodnikov and Danckwardt, 2021) bear great potential to decipher the resulting functional implications including the coupling of 3′end processing with genome integrity. Combined with technologies that enable the genome-wide interrogation of intermediates of impaired DNA damage repair inflict [such as R-loops; (Ginno et al., 2012; Yan et al., 2019; Wang et al., 2021)] or the mapping of the resulting consequences [dsDNA breaks etc., (Singh et al., 1988; Zhu et al., 2019)], complemented by loss-of-function studies, will help to further unwind the intricate reciprocal coupling between CPA and DDR. These studies are urgently needed to better understand the sequence of these events, their contribution to common pathologies, and to uncover novel potentially druggable driver lesions in devastating disorders such as cancer.

## Supplementary Material

Supplementary Table S1 Examples of DDR genes that are affected by alternative polyadenylation (APA) and DDR components affecting cleavage and polyadenylation.
