# Supplementary material for: The Bidirectional Link Between RNA Cleavage and Polyadenylation and Genome Stability: Recent Insights From a Systematic Screen
Source: Front Genet. 2022 Apr 28;13:854907. doi: 10.3389/fgene.2022.854907 (PMC9095915; doi:10.3389/fgene.2022.854907)
Supplement: Supplementary file 1 [file Table1.DOCX]

| **DDR Components** | | | | | | | |
| --- | --- | --- | --- | --- | --- | --- | --- |
| **Gene  symbol** | **Type** |  | **Level of  regulation** | **Treatment** | **Condition** | **PMID** | **Ref.** |
| ASCC3 | Regulated | Isoforms with opposite effects on transcription recovery | ALE | UV-C | DNA damage | 28215706 | (Williamson et al., 2017) |
| BARD1 | Regulator | Cstf50 associates with BARD1 to inhibit CPA | CPA | Hydroxyurea, UV | DNA damage | 11257228 | (Kleiman and Manley, 2001) |
| BARD1 | Regulator | Deadenylation of transcripts upon damage | CPA | UV | DNA damage | 20379136 | (Cevher et al., 2010) |
| BARD1 | Regulator | BRM-BARD1-BRCA1-Cstf50 complex prevents ALE | ALE | Oxidative stress | Oxidative stress | 27591253 | (Fontana et al., 2017) |
| BARD1 | Regulator |  | CPA | Melphalan | Human papillomavirus | 29596642 | (Nilsson et al., 2018) |
| BIK | Regulated | Star-PAP controls the 3′-end cleavage and polyadenylation of the BIK pre-mRNA in a gene specific manner | CPA | Etoposide | DNA damage | 22244330 | (Li et al., 2012) |
| BRCA1 | Regulator | Deadenylation of transcripts upon damage | CPA | UV | DNA damage | 20379136 | (Cevher et al., 2010) |
| BRCA1 | Regulator |  | CPA | Melphalan | Human papillomavirus | 29596642 | (Nilsson et al., 2018) |
| BRM | Regulator | BRM-BARD1-BRCA1-Cstf50 complex prevents ALE | ALE | Oxidative stress | Oxidative stress | 27591253 | (Fontana et al., 2017) |
| CCND1 | Regulated | Specific 3'UTR isoform effect on cell cycle | APA | CRISPR/Cas9 | Cancer | 29717174 | (Wang et al., 2018) |
| CCT5 | Regulated |  | APA | Cancer cells | Cancer | 28964783 | (Sun et al., 2017) |
| CDC6 | Regulated | 3'-UTR shortening activates this proto-oncogene | APA | 17 β-Estradiol | Cancer | 22977174 | (Akman et al., 2012) |
| CDK12 | Regulator | Loss of CDK12 can disrupt DNA repair by affecting DDR genes with long transcripts and a large number of exons | ALE | Cancer cells | Cancer | 28334900 | (Tien et al., 2017) |
| CDK12 | Regulator | CDK12 regulates DNA repair genes by suppressing intronic polyadenylation | IPA | Cancer cells | Cancer | 30487607 | (Dubbury et al., 2018) |
| CDK12 | Regulator | DDR genes are uniquely susceptible to CDK12inhibition | IPA | Cancer cells | Cancer | 30988284 | (Krajewska et al., 2019) |
| CDK12 | Regulator | CDK12 controls G1/S progression by regulating RNAPII processivity | IPA | Factor depletion | Cancer | 31347271 | (Chirackal Manavalan et al., 2019) |
| CDK12 | Regulator | Inhibition or loss of CDK12/CDK13 triggers intronic polyadenylation site cleavage that suppresses the expression of core DNA damage response proteins | IPA | CDK12 inhibitor | Cancer | 31668947 | (Quereda et al., 2019) |
| CDK12 | Regulator | Inhibition or loss of CDK12/CDK13 triggers intronic polyadenylation site cleavage that suppresses the expression of core DNA damage response proteins | IPA | Factor depletion | Cancer | 32917631 | (Fan et al., 2020) |
| CDK13 | Regulator | Inhibition or loss of CDK12/CDK13 triggers intronic polyadenylation site cleavage that suppresses the expression of core DNA damage response proteins | IPA | Factor depletion | Cancer | 32917631 | (Fan et al., 2020) |
| CDKN1A | Regulated | CTD phosphorylation at Ser2 is not required for p21 CIP1 transcription, mRNA cleavage, or polyadenylation. | CPA | Doxorubicin | DNA damage | 16510875 | (Gomes et al., 2006) |
| CDKN1A | Regulated | CPA is facilitated through histone phosphorylation | CPA | Factor depletion | DNA damage | 25813038 | (Lee et al., 2015) |
| CDKN1A | Regulated |  | IPA | UV-C | DNA damage | 27462460 | (Devany et al., 2016) |
| CENPN | Regulated | ALE maturation in response to topoisomerase inhibitors | ALE | Doxorubicin | Cancer | 24577238 | (Dutertre et al., 2014) |
| DHX36 | Regulator | Regulation of p53 through G-quadruplex structure | CPA | UV | DNA damage | 27940037 | (Newman et al., 2017) |
| DKC1 | Regulated | Deletion of PAS leads to telomere shortening | CPA | Inherited disease | Dyskeratosis congenita | 10.1002/jha2.165 |  |
| E2F1 | Regulated |  | IPA | UV-C | DNA damage | 27462460 | (Devany et al., 2016) |
| FAS | Regulated | Regulation of CPA through G-quadruplex structure | CPA | UV-C | DNA damage | 27940037 | (Newman et al., 2017) |
| GADD45A | Regulated | Regulation of CPA through G-quadruplex structure | CPA | UV-C | DNA damage | 27940037 | (Newman et al., 2017) |
| GLS | Regulated | ALE maturation in response to topoisomerase inhibitors | ALE | Doxorubicin | Cancer | 24577238 | (Dutertre et al., 2014) |
| H3 | Regulator | CPA is facilitated through histone phosphorylation | CPA | Factor depletion | DNA damage | 25813038 | (Lee et al., 2015) |
| HNRNPF/H | Regulator | Regulation of p53 through G-quadruplex structure | CPA | UV, Doxorubicin | DNA damage | 21289067 | (Decorsière et al., 2011) |
| HPV16 | Regulated |  | CPA | Melphalan | Human papillomavirus | 29596642 | (Nilsson et al., 2018) |
| HuR | Regulator | ALE maturation in response to topoisomerase inhibitors | ALE | Doxorubicin | Cancer | 24577238 | (Dutertre et al., 2014) |
| HUWE1 | Regulator | Ubiquitinates PCF11 resulting in loss of | APA | Cancer cells | Cancer | 31980388 | (Yang et al., 2020) |
| INTS1/INTS8 | Regulator |  | CPA | Inherited disease | Neurodevelopmental delay | 28542170 | (Oegema et al., 2017) |
| KIF1B | Regulated | ALE maturation in response to topoisomerase inhibitors | ALE | Doxorubicin | Cancer | 24577238 | (Dutertre et al., 2014) |
| MBD1 | Regulated | ALE maturation in response to topoisomerase inhibitors | ALE | Doxorubicin | Cancer | 24577238 | (Dutertre et al., 2014) |
| MDM2 | Regulated | CPA is facilitated through histone phosphorylation | CPA | Factor depletion | DNA damage | 25813038 | (Lee et al., 2015) |
| MDM4 | Regulated | 3'-UTR shortening and escape from miRNA repression | APA | Expression of the oncogenic KrasG12D allele from its endogenous promoter can be specifically induced in lung by Adeno-Cre (AdCre) virus-mediated removal of a transcription termination element flanked with LoxP sites | Cancer | 24532687 | (Okada et al., 2014) |
| MSL1 | Regulated | Full-length MSL1 mRNA upregulation protects cells from DNA damage-induced apoptosis | APA | Doxorubicin | Cancer | 34644577 | (Kunisky et al., 2021) |
| mtPAP | Regulator | The mutation prevents the enzyme from polyadenylating mt-mRNAs | CPA | Inherited disease | Spastic ataxia with optic atrophy | 25008111 | (Wilson et al., 2014) |
| p53 | Regulator | p53 inhibits mRNA 3′ processing | CPA | UV | DNA damage | 21383700 | (Nazeer et al., 2011) |
| p97 | Regulator | Interplay between Cstf50 and chromatin-bound proteins | Chromatin remodelling | UV | DNA damage | 29180510 | (Fonseca et al., 2018) |
| PIP5K1A | Regulator | Star-PAP and associated kinase control the 3′-end cleavage and polyadenylation of the BIK pre-mRNA in a gene specific manner | CPA | Etoposide | DNA damage | 22244330 | (Li et al., 2012) |
| POLH | Regulated | Loss of the short PolH transcript significantly sensitizes cancer cells to cisplatin treatment | APA | Cancer cells | Cancer | 31064846 | (Zhang et al., 2019) |
| POLR2K | Regulated | Upregulation of 3'UTR short isoform in cancer | APA | Cancer cells | Cancer | 22753024 | (Lin et al., 2012) |
| PPM1B | Regulated | ALE maturation in response to topoisomerase inhibitors | ALE | Doxorubicin | Cancer | 24577238 | (Dutertre et al., 2014) |
| PRKCD | Regulator | Star-PAP and associated kinase control the 3′-end cleavage and polyadenylation of the BIK pre-mRNA in a gene specific manner | CPA | Etoposide | DNA damage | 22244330 | (Li et al., 2012) |
| PTEN | Regulated | Polymerase specific 3'UTR isoform production | APA | Etoposide | Cancer | 28911096 | (Li et al., 2017) |
| PTPN6 | Regulated |  | APA | Oxidative stress | Cancer | 26725650 | (Batista et al., 2016) |
| PUMA | Regulated | Regulation of CPA through G-quadruplex structure | CPA | UV-C | DNA damage | 27940037 | (Newman et al., 2017) |
| RAB3IP | Regulated |  | APA | Cancer cells | Cancer | 28964783 | (Sun et al., 2017) |
| RFC3 | Regulated | ALE maturation in response to topoisomerase inhibitors | ALE | Doxorubicin | Cancer | 24577238 | (Dutertre et al., 2014) |
| RPRD1A | Regulated |  | ALE | Oxidative stress | Oxidative stress | 27591253 | (Fontana et al., 2017) |
| TMEM267 | Regulated |  | APA | Cancer cells | Cancer | 28964783 | (Sun et al., 2017) |
| TP53 | Regulated | Regulation of CPA through G-quadruplex structure | CPA | UV-C | DNA damage | 27940037 | (Newman et al., 2017) |
| UBA5 | Regulated |  | APA | Cancer cells | Cancer | 28964783 | (Sun et al., 2017) |
| ZEB1 | Regulated | 3'-UTR shortening and escape from miRNA repression | APA | Cancer cells | Cancer | 29120411 | (Passacantilli et al., 2017) |
|  | | | | | | | |
| **CPA Components** | | | | | | | |
| **Gene  symbol** | **Type** |  | **Level of  regulation** | **Treatment** | **Condition** | **PMID** | **Ref.** |
| CPSF160 | Regulator | Efficient CPA-mediated pre-mRNA cleavage confers replication stress resilience | CPA | Factor depletion | Cancer | 30639241 | (Teloni et al., 2019) |
| CPSF160 | Regulator | Cell cycle alteration | CPA | Factor depletion | Cancer | 32929364 | (Wang et al., 2020) |
| CPSF30 | Regulator |  | CPA | Melphalan | Human papillomavirus | 29596642 | (Nilsson et al., 2018) |
| CPSF30 | Regulator | Efficient CPA-mediated pre-mRNA cleavage confers replication stress resilience | CPA | Factor depletion | Cancer | 30639241 | (Teloni et al., 2019) |
| Cstf | Regulator | Deadenylation of transcripts upon damage | CPA | UV | DNA damage | 20379136 | (Cevher et al., 2010) |
| Cstf50 | Regulator | Cstf50 associates with BARD1 to inhibit CPA | CPA | Hydroxyurea, UV | DNA damage | 11257228 | (Kleiman and Manley, 2001) |
| Cstf50 | Regulator | BRM-BARD1-BRCA1-Cstf50 complex prevents ALE | ALE | Oxidative stress (paraquat, H2O2) | Oxidative stress | 27591253 | (Fontana et al., 2017) |
| Cstf50 | Regulator | Interplay between Cstf50 and chromatin-bound proteins | Chromatin remodelling | UV | DNA damage | 29180510 | (Fonseca et al., 2018) |
| ELAVL1 | Regulator | ALE maturation in response to topoisomerase inhibitors | ALE | Doxorubicin | Cancer | 24577239 | (Raptis et al., 2013) |
| FIP1L1 | Regulator | Efficient CPA-mediated pre-mRNA cleavage confers replication stress resilience | CPA | Factor depletion | Cancer | 30639241 | (Teloni et al., 2019) |
| PABN1 | Regulator | Mutation leads to interference with pre-mRNA processing | CPA | Inherited disease | Oculopharyngeal muscular dystrophy | 16756225 28011929 27152426 | (Riaz et al., 2016;Richard et al., 2017;Nilsson et al., 2018) |
| PABPN1 | Regulator | Cell cycle alteration | CPA | Factor depletion | Cancer | 32929364 | (Wang et al., 2020) |
| PARN | Regulator | Deadenylation of transcripts upon damage | CPA | UV | DNA damage | 20379136 | (Cevher et al., 2010) |
| PARN | Regulator | Early DNA damage response, G2/M arrest, and increased cell death | CPA | Inherited disease | Dyskeratosis congenita | 25893599 | (Tummala et al., 2015) |
| RNAPII | Regulator | Deadenylation of transcripts upon damage | CPA | UV | DNA damage | 20379136 | (Cevher et al., 2010) |
| RNAPII | Regulator | Isoforms with opposite effects on transcription recovery | ALE | UV-C | DNA damage | 28215706 | (Williamson et al., 2017) |
| StarPAP | Regulator | Star-PAP and associated kinase control the 3′-end cleavage and polyadenylation of the BIK pre-mRNA in a gene specific manner | CPA | Etoposide | DNA damage | 22244330 | (Li et al., 2012) |
| StarPAP | Regulator | Polymerase specific 3'UTR isoform production | APA | Etoposide | Cancer | 28911096 | (Li et al., 2017) |
| U1 snRNA | Regulator | IPA activation during DDR correlates with a decrease in U1 snRNA levels | IPA | UV-C | DNA damage | 27462460 | (Devany et al., 2016) |
| U1 snRNP | Regulator | IPA activation during DDR correlates with a decrease in U1 snRNA levels | IPA | UV-C | DNA damage | 27462460 | (Devany et al., 2016) |
| WDR33 | Regulator | Efficient CPA-mediated pre-mRNA cleavage confers replication stress resilience | CPA | Factor depletion | Cancer | 30639241 | (Teloni et al., 2019) |

Akman, B.H., Can, T., and Erson-Bensan, A.E. (2012). Estrogen-induced upregulation and 3'-UTR shortening of CDC6. *Nucleic Acids Res* 40**,** 10679-10688.

Batista, L., Bourachot, B., Mateescu, B., Reyal, F., and Mechta-Grigoriou, F. (2016). Regulation of miR-200c/141 expression by intergenic DNA-looping and transcriptional read-through. *Nat Commun* 7**,** 8959.

Cevher, M.A., Zhang, X., Fernandez, S., Kim, S., Baquero, J., Nilsson, P., Lee, S., Virtanen, A., and Kleiman, F.E. (2010). Nuclear deadenylation/polyadenylation factors regulate 3' processing in response to DNA damage. *Embo j* 29**,** 1674-1687.

Chirackal Manavalan, A.P., Pilarova, K., Kluge, M., Bartholomeeusen, K., Rajecky, M., Oppelt, J., Khirsariya, P., Paruch, K., Krejci, L., Friedel, C.C., and Blazek, D. (2019). CDK12 controls G1/S progression by regulating RNAPII processivity at core DNA replication genes. *EMBO Rep* 20**,** e47592.

Decorsière, A., Cayrel, A., Vagner, S., and Millevoi, S. (2011). Essential role for the interaction between hnRNP H/F and a G quadruplex in maintaining p53 pre-mRNA 3'-end processing and function during DNA damage. *Genes Dev* 25**,** 220-225.

Devany, E., Park, J.Y., Murphy, M.R., Zakusilo, G., Baquero, J., Zhang, X., Hoque, M., Tian, B., and Kleiman, F.E. (2016). Intronic cleavage and polyadenylation regulates gene expression during DNA damage response through U1 snRNA. *Cell Discov* 2**,** 16013.

Dubbury, S.J., Boutz, P.L., and Sharp, P.A. (2018). CDK12 regulates DNA repair genes by suppressing intronic polyadenylation. *Nature* 564**,** 141-145.

Dutertre, M., Chakrama, F.Z., Combe, E., Desmet, F.O., Mortada, H., Polay Espinoza, M., Gratadou, L., and Auboeuf, D. (2014). A recently evolved class of alternative 3'-terminal exons involved in cell cycle regulation by topoisomerase inhibitors. *Nat Commun* 5**,** 3395.

Fan, Z., Devlin, J.R., Hogg, S.J., Doyle, M.A., Harrison, P.F., Todorovski, I., Cluse, L.A., Knight, D.A., Sandow, J.J., Gregory, G., Fox, A., Beilharz, T.H., Kwiatkowski, N., Scott, N.E., Vidakovic, A.T., Kelly, G.P., Svejstrup, J.Q., Geyer, M., Gray, N.S., Vervoort, S.J., and Johnstone, R.W. (2020). CDK13 cooperates with CDK12 to control global RNA polymerase II processivity. *Sci Adv* 6.

Fonseca, D., Baquero, J., Murphy, M.R., Aruggoda, G., Varriano, S., Sapienza, C., Mashadova, O., Rahman, S., and Kleiman, F.E. (2018). mRNA Processing Factor CstF-50 and Ubiquitin Escort Factor p97 Are BRCA1/BARD1 Cofactors Involved in Chromatin Remodeling during the DNA Damage Response. *Mol Cell Biol* 38.

Fontana, G.A., Rigamonti, A., Lenzken, S.C., Filosa, G., Alvarez, R., Calogero, R., Bianchi, M.E., and Barabino, S.M. (2017). Oxidative stress controls the choice of alternative last exons via a Brahma-BRCA1-CstF pathway. *Nucleic Acids Res* 45**,** 902-914.

Gomes, N.P., Bjerke, G., Llorente, B., Szostek, S.A., Emerson, B.M., and Espinosa, J.M. (2006). Gene-specific requirement for P-TEFb activity and RNA polymerase II phosphorylation within the p53 transcriptional program. *Genes Dev* 20**,** 601-612.

Kleiman, F.E., and Manley, J.L. (2001). The BARD1-CstF-50 interaction links mRNA 3' end formation to DNA damage and tumor suppression. *Cell* 104**,** 743-753.

Krajewska, M., Dries, R., Grassetti, A.V., Dust, S., Gao, Y., Huang, H., Sharma, B., Day, D.S., Kwiatkowski, N., Pomaville, M., Dodd, O., Chipumuro, E., Zhang, T., Greenleaf, A.L., Yuan, G.C., Gray, N.S., Young, R.A., Geyer, M., Gerber, S.A., and George, R.E. (2019). CDK12 loss in cancer cells affects DNA damage response genes through premature cleavage and polyadenylation. *Nat Commun* 10**,** 1757.

Kunisky, A.K., Anyaeche, V.I., Herron, R.S., Park, C.Y., and Hwang, H.W. (2021). Shift in MSL1 alternative polyadenylation in response to DNA damage protects cancer cells from chemotherapeutic agent-induced apoptosis. *Cell Rep* 37**,** 109815.

Lee, J.H., Kang, B.H., Jang, H., Kim, T.W., Choi, J., Kwak, S., Han, J., Cho, E.J., and Youn, H.D. (2015). AKT phosphorylates H3-threonine 45 to facilitate termination of gene transcription in response to DNA damage. *Nucleic Acids Res* 43**,** 4505-4516.

Li, W., Laishram, R.S., Ji, Z., Barlow, C.A., Tian, B., and Anderson, R.A. (2012). Star-PAP control of BIK expression and apoptosis is regulated by nuclear PIPKIα and PKCδ signaling. *Mol Cell* 45**,** 25-37.

Li, W., Li, W., Laishram, R.S., Hoque, M., Ji, Z., Tian, B., and Anderson, R.A. (2017). Distinct regulation of alternative polyadenylation and gene expression by nuclear poly(A) polymerases. *Nucleic Acids Res* 45**,** 8930-8942.

Lin, Y., Li, Z., Ozsolak, F., Kim, S.W., Arango-Argoty, G., Liu, T.T., Tenenbaum, S.A., Bailey, T., Monaghan, A.P., Milos, P.M., and John, B. (2012). An in-depth map of polyadenylation sites in cancer. *Nucleic Acids Res* 40**,** 8460-8471.

Nazeer, F.I., Devany, E., Mohammed, S., Fonseca, D., Akukwe, B., Taveras, C., and Kleiman, F.E. (2011). p53 inhibits mRNA 3' processing through its interaction with the CstF/BARD1 complex. *Oncogene* 30**,** 3073-3083.

Newman, M., Sfaxi, R., Saha, A., Monchaud, D., Teulade-Fichou, M.P., and Vagner, S. (2017). The G-Quadruplex-Specific RNA Helicase DHX36 Regulates p53 Pre-mRNA 3'-End Processing Following UV-Induced DNA Damage. *J Mol Biol* 429**,** 3121-3131.

Nilsson, K., Wu, C., Kajitani, N., Yu, H., Tsimtsirakis, E., Gong, L., Winquist, E.B., Glahder, J., Ekblad, L., Wennerberg, J., and Schwartz, S. (2018). The DNA damage response activates HPV16 late gene expression at the level of RNA processing. *Nucleic Acids Res* 46**,** 5029-5049.

Oegema, R., Baillat, D., Schot, R., Van Unen, L.M., Brooks, A., Kia, S.K., Hoogeboom, A.J.M., Xia, Z., Li, W., Cesaroni, M., Lequin, M.H., Van Slegtenhorst, M., Dobyns, W.B., De Coo, I.F.M., Verheijen, F.W., Kremer, A., Van Der Spek, P.J., Heijsman, D., Wagner, E.J., Fornerod, M., and Mancini, G.M.S. (2017). Human mutations in integrator complex subunits link transcriptome integrity to brain development. *PLoS Genet* 13**,** e1006809.

Okada, N., Lin, C.P., Ribeiro, M.C., Biton, A., Lai, G., He, X., Bu, P., Vogel, H., Jablons, D.M., Keller, A.C., Wilkinson, J.E., He, B., Speed, T.P., and He, L. (2014). A positive feedback between p53 and miR-34 miRNAs mediates tumor suppression. *Genes Dev* 28**,** 438-450.

Passacantilli, I., Panzeri, V., Bielli, P., Farini, D., Pilozzi, E., Fave, G.D., Capurso, G., and Sette, C. (2017). Alternative polyadenylation of ZEB1 promotes its translation during genotoxic stress in pancreatic cancer cells. *Cell Death Dis* 8**,** e3168.

Quereda, V., Bayle, S., Vena, F., Frydman, S.M., Monastyrskyi, A., Roush, W.R., and Duckett, D.R. (2019). Therapeutic Targeting of CDK12/CDK13 in Triple-Negative Breast Cancer. *Cancer Cell* 36**,** 545-558.e547.

Raptis, V., Georgianos, P.I., Sarafidis, P.A., Sioulis, A., Makedou, K., Makedou, A., Grekas, D.M., and Kapoulas, S. (2013). Elevated asymmetric dimethylarginine is associated with oxidant stress aggravation in patients with early stage autosomal dominant polycystic kidney disease. *Kidney Blood Press Res* 38**,** 72-82.

Riaz, M., Raz, Y., Van Putten, M., Paniagua-Soriano, G., Krom, Y.D., Florea, B.I., and Raz, V. (2016). PABPN1-Dependent mRNA Processing Induces Muscle Wasting. *PLoS Genet* 12**,** e1006031.

Richard, P., Trollet, C., Stojkovic, T., De Becdelievre, A., Perie, S., Pouget, J., and Eymard, B. (2017). Correlation between PABPN1 genotype and disease severity in oculopharyngeal muscular dystrophy. *Neurology* 88**,** 359-365.

Sun, M., Ding, J., Li, D., Yang, G., Cheng, Z., and Zhu, Q. (2017). NUDT21 regulates 3'-UTR length and microRNA-mediated gene silencing in hepatocellular carcinoma. *Cancer Lett* 410**,** 158-168.

Teloni, F., Michelena, J., Lezaja, A., Kilic, S., Ambrosi, C., Menon, S., Dobrovolna, J., Imhof, R., Janscak, P., Baubec, T., and Altmeyer, M. (2019). Efficient Pre-mRNA Cleavage Prevents Replication-Stress-Associated Genome Instability. *Mol Cell* 73**,** 670-683.e612.

Tien, J.F., Mazloomian, A., Cheng, S.G., Hughes, C.S., Chow, C.C.T., Canapi, L.T., Oloumi, A., Trigo-Gonzalez, G., Bashashati, A., Xu, J., Chang, V.C., Shah, S.P., Aparicio, S., and Morin, G.B. (2017). CDK12 regulates alternative last exon mRNA splicing and promotes breast cancer cell invasion. *Nucleic Acids Res* 45**,** 6698-6716.

Tummala, H., Walne, A., Collopy, L., Cardoso, S., De La Fuente, J., Lawson, S., Powell, J., Cooper, N., Foster, A., Mohammed, S., Plagnol, V., Vulliamy, T., and Dokal, I. (2015). Poly(A)-specific ribonuclease deficiency impacts telomere biology and causes dyskeratosis congenita. *J Clin Invest* 125**,** 2151-2160.

Wang, L., Lang, G.T., Xue, M.Z., Yang, L., Chen, L., Yao, L., Li, X.G., Wang, P., Hu, X., and Shao, Z.M. (2020). Dissecting the heterogeneity of the alternative polyadenylation profiles in triple-negative breast cancers. *Theranostics* 10**,** 10531-10547.

Wang, Q., He, G., Hou, M., Chen, L., Chen, S., Xu, A., and Fu, Y. (2018). Cell Cycle Regulation by Alternative Polyadenylation of CCND1. *Sci Rep* 8**,** 6824.

Williamson, L., Saponaro, M., Boeing, S., East, P., Mitter, R., Kantidakis, T., Kelly, G.P., Lobley, A., Walker, J., Spencer-Dene, B., Howell, M., Stewart, A., and Svejstrup, J.Q. (2017). UV Irradiation Induces a Non-coding RNA that Functionally Opposes the Protein Encoded by the Same Gene. *Cell* 168**,** 843-855.e813.

Wilson, W.C., Hornig-Do, H.T., Bruni, F., Chang, J.H., Jourdain, A.A., Martinou, J.C., Falkenberg, M., Spåhr, H., Larsson, N.G., Lewis, R.J., Hewitt, L., Baslé, A., Cross, H.E., Tong, L., Lebel, R.R., Crosby, A.H., Chrzanowska-Lightowlers, Z.M., and Lightowlers, R.N. (2014). A human mitochondrial poly(A) polymerase mutation reveals the complexities of post-transcriptional mitochondrial gene expression. *Hum Mol Genet* 23**,** 6345-6355.

Yang, S.W., Li, L., Connelly, J.P., Porter, S.N., Kodali, K., Gan, H., Park, J.M., Tacer, K.F., Tillman, H., Peng, J., Pruett-Miller, S.M., Li, W., and Potts, P.R. (2020). A Cancer-Specific Ubiquitin Ligase Drives mRNA Alternative Polyadenylation by Ubiquitinating the mRNA 3' End Processing Complex. *Mol Cell* 77**,** 1206-1221.e1207.

Zhang, J., Sun, W., Ren, C., Kong, X., Yan, W., and Chen, X. (2019). A PolH Transcript with a Short 3'UTR Enhances PolH Expression and Mediates Cisplatin Resistance. *Cancer Res* 79**,** 3714-3724.
